# Pregnant women’s experiences of routine counselling and testing for HIV in Eastern Uganda: a qualitative study

**DOI:** 10.1186/1472-6963-13-189

**Published:** 2013-05-24

**Authors:** Joseph Rujumba, Stella Neema, James K Tumwine, Thorkild Tylleskär, Harald K Heggenhougen

**Affiliations:** 1 Department of Paediatrics and Child Health, College of Health Sciences, Makerere University, PO Box 7072, Kampala, Uganda; 2 Centre for International Health, University of Bergen, Bergen, Norway; 3 Department of Sociology and Anthropology, College of Humanities and Social Sciences, Makerere University, Kampala, Uganda

## Abstract

**Background:**

Routine HIV counselling and testing as part of antenatal care has been institutionalized in Uganda as an entry point for pregnant women into the prevention of mother-to-child transmission of HIV (PMTCT) programme. Understanding how women experience this mode of HIV testing is important to generate ideas on how to strengthen the PMTCT programme. We explored pregnant HIV positive and negative women’s experiences of routine counselling and testing in Mbale District, Eastern Uganda and formulated suggestions for improving service delivery.

**Methods:**

This was a qualitative study conducted at Mbale Regional Referral Hospital in Eastern Uganda between January and May 2010. Data were collected using in-depth interviews with 30 pregnant women (15 HIV positive and 15 HIV negative) attending an antenatal clinic, six key informant interviews with health workers providing antenatal care and observations. Data were analyzed using a content thematic approach.

**Results:**

Prior to attending their current ANC visit, most women knew that the hospital provided HIV counselling and testing services as part of antenatal care (ANC). HIV testing was perceived as compulsory for all women attending ANC at the hospital but beneficial, for mothers, especially those who test HIV positive and their unborn babies. Most HIV positive women were satisfied with the immediate counselling they received from health workers, but identified the need to provide follow up counselling and support after the test, as areas for improvement. However, most HIV negative women mentioned that they were given inadequate attention during post-test counselling. This left them with unanswered questions and, for some, doubts about the negative test results.

**Conclusions:**

In this setting, routine HIV counselling and testing services are known and acceptable to mothers. There is need to strengthen post-test and follow up counselling for both HIV positive and negative women in order to maximize opportunities for primary and post exposure HIV prevention. Partnerships and linkages with people living with HIV, especially those in existing support groups such as those at The AIDS Support Organization (TASO), may help to strengthen counselling and support for pregnant women. For effective HIV prevention, women who test HIV negative should be supported to remain negative.

## Background

In Uganda, the programme for prevention of mother-to-child transmission of HIV (PMTCT) was initiated in 2000, originally using the voluntary counselling and testing (VCT) approach. This was later changed to routine counselling and testing for HIV (RCT), which has been institutionalized in Uganda since 2006 [1]. Under VCT, the initiative to test is by the client while for RCT, providers initiate the test but clients can opt not to take the test. Thus, for PMTCT, HIV counselling and testing became integrated into antenatal clinics (ANC), childbirth and postpartum health care services [2]. The proportion of health facilities providing PMTCT services in Uganda has increased rapidly in recent years. However, the utilisation of these services remains insufficient. The proportion of pregnant women living with HIV in Uganda who received antiretroviral drugs for preventing mother-to-child transmission of HIV was estimated at 51.6% in 2009 [3].

Routine HIV testing has been part of the standard of care in many high income countries since the late 1980s and early 1990s. For instance, HIV testing has been offered as part of routine antenatal care to all women in Sweden and Norway [4,5] since 1987 and in France [6,7] since 1993. However, routine HIV testing as part of standard care is relatively new in most African countries [8], introduced following the initiation of the Joint United Nations Programme on HIV/AIDS (UNAIDS) and World Health Organisation (WHO) recommendations in 2004 [9]. In part, routine testing was a response to the low HIV testing rates under the VCT approach but also the need to increase the number of people benefiting from the increased availability of HIV treatment and prevention interventions including the PMTCT programme [10-12]. Routine counselling has been associated with increased testing rates especially among women attending ANC in many African settings [10,11,13-15]. For instance, in 2010, (with RCT as a dominant approach), it was estimated that 63% of all pregnant women in Uganda tested for HIV, a dramatic increase from 18% in 2005 under VCT [16] The new HIV testing approach is likely to be associated with unique experiences by women who go through it, especially, given the limited coverage of HIV testing in the general Ugandan population, estimated at only 20% [3]. Understanding women’s experiences of routine HIV counselling and testing is important given the high HIV prevalence in the country currently in 2013, estimated at 7.3% [17] and a shortage of health workers [18,19]. More so, there is limited information on how routine provision of HIV testing is perceived and experienced by women in low income countries. Some studies have indicated that HIV testing is perceived as mandatory [20-22] and women do not fully understand the reasons for being tested [21]. Understanding how women perceive and experience HIV testing as part of antenatal care in a high HIV prevalence setting is important to generate ideas on how to strengthen the PMTCT programme and ensuring that HIV counselling, follow-up support and prevention interventions are tailored to the needs of both HIV positive and negative women.

In this paper, we draw on Critical Theory [23] and locate women’s experiences of routine HIV counselling and testing within the broader socio-structural context and processes in which these services are delivered and experienced by HIV negative and positive women. Showing how people make different but valid sense of experiences makes critical theory a rational framework for this paper. From a critical theory perspective, discussion of health problems, separate from their social context, only serves to downplay social relationships underlying such problems [23]. On the contrary, the very core of Critical Theory involves paying attention to social structures and relationships at micro and macro levels of society that contribute to the pattern of human behaviour, beliefs and attitudes [24]. In our case Critical Theory is suitable to explain how women’s experiences of routine HIV testing relate to the broader social context in which they lead their lives. At macro level, this context includes international and national HIV guidelines and how these are implemented by health facilities often characterised by stock-out of medical supplies and few staff. At micro level factors like gender and power relations, women’s past interface with formal and informal sources of information, like previous antenatal care experiences or interactions with family members and friends who have accessed health services in the study setting. Critical Theory stems from the assumption that psychological theories (of behaviour)often conceal complex political, economic and social relationships in society which constrain individuals and shape behaviour [24]. We argue in this paper that, women’s experiences of routine HIV counselling and testing and the implications of these experiences on prevention of mother to child transmission of HIV in Uganda, are in part a reflection of broader structural facilitators and constraints at family, community, health systems as well as the national and global policy levels. It is against this background that we explored pregnant HIV positive and negative women’s experiences of routine counselling and testing in Mbale District, Eastern Uganda, and formulated suggestions for improving service delivery.

## Methods

### Study area

The study was conducted at the antenatal clinic, Mbale Regional Referral Hospital, about 245 kilometres East of Kampala, the capital city of Uganda. The district has a population of 416,600 [25], with the vast majority residing in rural areas [26]. Mbale Regional Referral Hospital has an estimated tertiary catchment population of 1.9 million people [14] from Mbale and the neighbouring districts in eastern Uganda.

Antenatal care services at the hospital are provided daily on weekdays by an average staff of five health workers, mostly midwives. On average, 60 pregnant women attend the antenatal clinic daily. Over half of these are new attendees requiring HIV counselling and testing. All antenatal attendees are given HIV education/group pre-test counselling at the start of each clinic session, covering maternal and newborn care as well as HIV-specific areas such as modes of HIV transmission, prevention, the need to undergo HIV testing, PMTCT and positive living in case one is HIV positive.

Health education talks are conducted daily, on average lasting for one hour. Talks are highly interactive involving health workers and antenatal attendees. Health workers provide information, but also solicit for responses from ANC attendees to build on what women already know about HIV and maternal and new-born heath before attending the clinic. The health education talks are conducted from the waiting area within the antenatal clinic by health workers on rotational basis. Whereas it is possible that the presence of the researchers during health education talks could have affected the quality and length of the talks, no major variations were observed in talks done at the start of the study and those at the end. Thus the influence of the researchers could have been minimal. The aim of the pre-test session is to prepare the women for the HIV test [1]. At the end of a pre-test session, new antenatal attendees move individually to the testing room located within the antenatal clinic to take the HIV test. At Mbale hospital, in line with the Uganda national guidelines, a sequential HIV testing algorithm, with same-day results is used. The testing algorithm includes three rapid tests used on one blood sample. ‘Determine’ is used for first screening, STAT-PAK for a second test and Uni-Gold as a ‘tie-breaker’ test. An individual is classified as HIV –uninfected if ‘Determine’ results are negative, or HIV infected if test results of both ‘Determine’ and STAT-PAK are positive. In case of discordant test results of ‘Determine’ and STAT-PAK, samples are tested using UNIGOLD. HIV results are given on the same day in an individual post-test counselling session conducted within the antenatal clinic. A previous study conducted in the same setting established that 99.5% of the antenatal attendees were tested for HIV [27]. All health workers were trained in PMTCT and routine HIV counselling and testing.

The hospital was selected because it serves a rural population and it is one of the oldest PMTCT sites in Uganda. The PMTCT programme at Mbale Hospital started in May 2002 with HIV testing being offered under the voluntary counselling and testing (VCT) approach. In 2006, Mbale Hospital started providing HIV testing for PMTCT as a routine service integrated within antenatal and child birth clinics.

### Study design

A qualitative research design was adopted to provide an in-depth understanding of the experiences of pregnant women with antenatal-based HIV testing [28] and the meaning mothers attach to this experience [29]. This is relevant for our understanding of both the process and the outcome of health care policies and initiatives in health promotion and prevention [30].

### Study participants and sampling

A total of 30 pregnant women (15 HIV positive and 15 HIV negative) attending follow up antenatal clinics at Mbale Regional Referral Hospital participated in the study from January to May 2010. Women were interviewed on their subsequent ANC visit after the initial visit when they received HIV counselling and testing during the current pregnancy. Variations in age, parity and education level were considered in the selection of study participants. Inclusion in the study was based on consent to participate, being pregnant, and having taken an HIV test on their previous antenatal visit. All study participants were aged 18 years or over. Eligible women were identified in cooperation with the health workers involved in antenatal care, thus facilitating access to study participants [29]. The identified women were referred to members of the study team stationed at the antenatal clinic. The researchers explained the purpose of the study and obtained consent. The study participants were enrolled consecutively after undergoing their routine consultation and assessment. Interviewing continued until we felt that the information from later interviews did not differ from earlier interviews. Six health workers selected purposively on the basis of being involved in routine HIV counselling and testing participated in the study as key informants. These included one doctor, two counsellors and three nurse midwives.

### Individual interviews with pregnant women

Semi-structured interviews [31] were conducted to elicit information on women’s experiences with routine HIV counselling and testing as part of antenatal care. Individual interviews were used since HIV is still a sensitive and stigmatizing condition. A pre-tested interview guide [32,33] was used to explore the pregnant women’s experiences with RCT and consisted of structured questions on women’s characteristics and open-ended qualitative questions on: prior knowledge about RCT provision, experiences with RCT, meaning of HIV test results, the conduct of health workers and suggestions to improve HIV counselling and testing. The interview guide was flexible with probes to allow an in-depth understanding of women’s experiences of the RCT programme as an entry point into the PMTCT programme and allowed women to identify areas requiring improvement from their own perspective as service users.

Interviews lasted for about 40–45 minutes and most interviews (27) were audio recorded; the exceptions were three women (one HIV positive and two HIV negative) who did not consent to audio recording. Interviewers were paired, one asking questions and the other taking notes. Interviews were conducted in *Lumasaba*, *Luganda* and a few in English. The first author conducted interviews in English and *Luganda* and was assisted by three female research assistants, all university graduates and with experience in conducting qualitative interviews. Audio-recorded interviews were transcribed and translated into English by a research assistant.

### Key informant interviews

A key informant interview guide was used to conduct the interviews. The interviews explored health workers experience with RCT, the process, what they felt were women’s concerns about the RCT and what needs to be done to improve the programme.

#### Observations during health education talks

To gain more understanding of the context within which routine HIV counselling and testing services were offered, the first author and research assistants attended and made observations during health education talks and the process of HIV counselling and testing. These observations were not structured. Individual researchers noted observations and shared them in research team meetings held at the end of each day of data collection. The health education talks were not audio recorded.

### Data analysis

Preliminary data analysis was concurrent with data collection. At the end of each day of data collection, a research team meeting was held to share emerging issues and identify areas for further data collection. The first author de-briefed all co-authors on preliminary insights and emerging issues from the study.

Further data analysis was conducted by the first author in close collaboration with the last author. The transcripts were exported to NVivo [34] version 9.0 and analyzed using a content thematic approach [35]. The analysis involved multiple readings of interview scripts to understand the data and to identify new themes and refine those in the interview guide. Data was then grouped under themes and sub-themes for interpretation. During the coding process, quotations illustrative of both the common and minority perspectives of women’s experiences of HIV testing as part of antenatal care were identified and are used in the presentation of the study findings. Concurrent triangulation was conducted which involved the analysis of findings from pregnant women and those from key informants at the same time to identify areas of agreement and disagreement. In addition, we conducted sub-group analysis of HIV positive and negative women.

### Ethical considerations

Ethical approval for the study was obtained from the Uganda National Council for Science and Technology, Makerere University, College of Health Sciences, Research and Ethics Committee and The Mbale Regional Referral Hospital Institutional Review Committee. Permission was also obtained from the management of Mbale Hospital and the Mbale District administration. All study participants provided written consent, they were assured of confidentiality and each interview was conducted in a separate room provided by the antenatal clinic management. Research assistants were trained on the approach to data collection and the ethical issues involved in HIV research.

## Results

### Characteristics of study participants

The ages of the 30 study participants ranged from 18 to 43 years. Most of the women had previously given birth; half had attained primary education, most of them were married and depended on subsistence agriculture for a living. The major themes that emerged from the interviews were: (1) HIV testing as part of ANC was no surprise, (2) HIV testing was compulsory for all pregnant women, (3) HIV testing was a difficult but beneficial step, (4) HIV test results meant a mixture of joy and sorrow, (5) post-test counselling was insufficient, and (6) counsellors were supportive but constrained because of time (Table 1).

**Table 1:** Thematic presentation of pregnant women’s experiences of HIV testing as part of antenatal care at Mbale Hospital

**Organising themes**	**Sub-themes**	**HIV negative women**	**HIV positive women**
• HIV testing as part of ANC was no surprise	• HIV testing as part of ANC was no surprise	Yes	Yes
• HIV testing was compulsory for all pregnant women	• HIV testing was for all pregnant women	Yes	Yes
• Testing was a difficult step but beneficial	• Fear of a positive test	Yes	Yes
	• Testing is beneficial to baby and mother	Yes	Yes
• Insufficient post-test counselling	• Health education session was educative and informative	Yes	Yes
	• Limited time for post-test	Yes	No
	• Post-test session adequate	No	Yes
• Test results - a mix of joy and sorrow	• Negative results sign of faithfulness	Yes	No
	• Relief from stress and fear	Yes	No
	• Doubt about result	Yes	Yes
	• Desire to remain negative	Yes	No
	• Partner unfaithfulness and betrayal	No	Yes
	• Uncertain future and survival	No	Yes
	• Determination to live and protect child	No	Yes
• Staff supportive but constrained	• Staff supportive and caring	Yes	Yes
	• Staff overwhelmed by many women	Yes	Yes

### HIV testing as part of ANC was no surprise

Generally, most women were aware that routine HIV counselling and testing was provided at Mbale Hospital prior to their coming to the hospital. Overall, only one study participant, who was a student, was not aware that the hospital offered HIV testing as part of antenatal care. Most women had learnt about the provision of HIV testing at Mbale Hospital through friends, radio, health workers at lower level health facilities, and a few from attending at the same hospital previously:

I knew that HIV counselling and testing services are provided in all health centres and hospitals, I tested here in my previous pregnancy… (Married, 30 years, HIV positive).

Those who had attended antenatal care at the lower level health centres observed that HIV testing services exist at those health centres, though infrequently, owing to shortage of HIV test kits, as one woman noted:

I knew that these days all women when they are pregnant they are tested. I first attended antenatal at Namatala Health Centre, the nurse told us that we should test but she did not have what to use to test us. We were about 10 women so if their laboratory was working, I would have tested from there. We also get information about HIV testing from radios and our friends who have tested (Married, 32 years, HIV negative).

The above narratives of women and indeed those of health workers indicate that most women had prior information from both formal and informal sources before going for the antenatal visit, and this prepared them to expect HIV counselling and testing at the antenatal clinic. The information provided by health workers on the need to test, emphasized women’s expectation of an HIV test.

### HIV testing was perceived as compulsory for all pregnant women

Most of the study participants understood HIV testing as part of antenatal care as compulsory for all pregnant women.

These days, when you go for pregnancy check-up, health workers test all women for HIV. It started like three years ago… These days that is how it is at all health centres. Even radio announcements have been made informing women to test for HIV when pregnant (Single, 20 years, HIV positive).

Women’s perception of HIV testing as compulsory was strengthened by the practices at health facilities and the ongoing discourse in support of the PMTCT programme especially, thought the media where health workers and other government officials often disseminate information and call upon pregnant women to go for HIV testing. Some women perceived HIV testing for pregnant women as a new government “law” aimed at protecting babies from HIV infection:

The government introduced a new law to test all pregnant women for HIV so that those found to have HIV they can be given drugs to save their babies. It is for every pregnant woman… (Married, 28 years, HIV positive).

The implication here is that the high number of women accepting to test for HIV in the study setting may in part be an indication of women’s compliance with this new “government law”. Interviews with health workers and observations at the clinic revealed that whereas women had an option of opting out of HIV testing, health workers emphasized the benefits of HIV testing to the mothers during pre-test counselling; the possibility of opt out was seldom discussed, as one of the health workers noted:

Testing is like forcing in a good way. We tell the mothers the benefits for them and their babies like PMTCT if they are HIV positive, accessing treatment for themselves and remaining HIV negative for those who are found HIV negative. Then the mothers choose for themselves…. (Health worker).

Some health workers revealed that, practicing opt out was in conflict with the known benefits of HIV testing as illustrated in the following quote.

It is really hard to tell mothers you have a choice not to test when we know all the benefits of testing including PMTCT…. Even those mothers who would have wanted not to test, they test because they see every other person is testing… (Health worker).

The narratives of women and health workers reveal that the health facility practice where all ANC attendees are tested for HIV and health workers’ emphasis on the benefits of HIV testing during health education and counselling sessions served to confirm women’s perception of routine HIV counselling and testing as a compulsory service.

### HIV testing was a difficult – but beneficial step

Some HIV positive and HIV negative women described moving forward for the test as a “difficult step”. Common among the narratives of women was that they had to overcome fear of a likely positive HIV test.

When my turn came to move to the desk where blood was being taken from, it was hard. Many questions were running in my head, what if I am found to have the virus (HIV) what will I do to live longer and care for my children? Won’t my husband say I am the cause?… but since it was for everyone I went for it (HIV test). Thank God I was found negative (Married, 32 years, HIV negative).

The difficulties of going forward for an HIV test among most women were related to the fear of a positive HIV test result which threatened normal life and triggered thoughts about the possibility of being blamed by their sexual partners for bringing HIV infection into the family and the challenges of living with HIV. For some women who experienced an “inner” struggle about HIV testing, they gained the courage to go for the test since all of them who attended ANC and had never tested for HIV went for the test.

For me at the beginning I had fears within myself. But since every woman was going for it (HIV testing), I had to go. I asked myself, if I do not go for the test, what if I have the virus, I would infect my baby so I went for it… (Married, 24 years, HIV negative).

The above narrative of a HIV negative woman indicates that the strong desire of women to save their babies from HIV infection in case they tested HIV positive encouraged them to overcome the fear and go for HIV testing.

Another important theme that emerged in the narratives of most women in our study was the understanding that HIV testing within the antenatal clinic was beneficial to women and their children, especially if one tested HIV positive. The benefits mentioned included access to treatment, guidance on positive living and the package for PMTCT. In view of this, one of the women explained:

I don’t blame the health workers because they want to protect our babies and those who are found HIV positive they get treatment to live longer (Married, 20 years, HIV positive).

Similarly, another woman who tested HIV negative added:

HIV testing is good. If I have HIV, my child should be saved from it because children are innocent (Single, 18 years, HIV negative).

Throughout the interviews, most women believed that routine counselling and testing was a good policy as it provided them with an opportunity to save their babies from HIV infection in case they were found to be HIV positive. Underlying the general acceptance of routine testing were the values attached to children and the moral imperative that children were innocent and thus should be protected from HIV infection.

For some HIV positive women, HIV counselling and testing had helped them to appreciate the role of HIV treatment.

I would recommend any person to take such a decision (test for HIV)… knowing my status has helped me, I now know that I need to start treatment. I don’t think I will die of HIV soon. I have a friend who is in TASO and she is looking very good, those drugs work because before she started the drugs she was almost gone! (Married, 40 years, HIV positive).

For this woman, her experience of HIV testing stimulated interest in HIV treatment which was further strengthened by knowing someone whose life had improved due to ARVs, an indication that individual women’s experiences of HIV testing are part of their wider experiences within the society where they live. Overall, the benefits of HIV testing for the women who test HIV negative were rarely mentioned by women, indicating a major gap for HIV prevention.

### The central place of health education

Throughout the interviews, women reiterated how they had been assisted by the health education talk they received before testing. Many women mentioned that the health education session had enlightened them more about the importance of HIV testing and prepared them to undergo the test. The need to practice fidelity among women who test HIV negative, HIV testing as an entry point into the PMTCT programme and the process and likely outcome of the HIV test were other key aspects emphasised during health education talks mentioned by women.

The nurse explained to us that we have to test for HIV so that we know how we are and those found HIV positive, they can be given the drugs to protect their babies from HIV and those who will be found not having HIV should avoid getting it by remaining faithful to their partners (Married, 28 years, HIV negative).

Whereas being faithful was a key message emphasised in health education talks by health workers, it is important to note that fidelity must involve men as well as women if it is to be effective in HIV prevention.

For some women who tested HIV positive, the messages they had received during the health education sessions which doubled as pre-test counselling sessions were a source of reminder and comfort whenever the realities of a positive HIV diagnosis became real in their lives.

I did not expect to have HIV, but I remembered the counsellor had said when educating us that every one is at risk but these days drugs are available and one can live for many years even with HIV that made me feel strong (Married, 28 years, HIV positive).

Indeed, observations during the ANC clinic confirmed that women who attended ANC at Mbale Hospital are taken through a detailed and interactive health education talk emphasizing danger signs during pregnancy, the importance of HIV testing, infant feeding and the fact that all pregnant women should test for HIV.

### Women’s understanding of HIV test results – a mixture of joy and sorrow

Women in our study expressed various interpretations and understanding of HIV test results. For most women who tested HIV negative the results meant joy while for most of the women who tested HIV positive the results were associated with shock, worry and sorrow. Study participants who were HIV positive revealed that their test results meant uncertainty about the future, and betrayal and infidelity by their sexual partners, as one of the women noted:

I received my results and I was upset because I have been married for 20 years without ever moving out of marriage… my husband had betrayed me. I cried while at the hospital but the nurse comforted me. When I reached home, I would find myself crying… (Married, 43 years, HIV positive).

As indicated above, most women who had tested HIV positive associated their HIV status to infidelity by their partners. Some women equated HIV positive results with “death” despite the fact that ARVs are becoming increasingly available.

When I was told I have HIV I thought for a while… I knew this man had killed me (Married, 19 years, HIV positive).

The phrases “this man had killed me” and ‘my husband had betrayed me’ are expressions that the women had contracted HIV infection from their partners, a belief that was common among HIV positive women. These findings in part reflect the vulnerability of women to HIV infection, even when they themselves may be faithful.

For most women who tested HIV positive, concerns about the future and care for their children dominated the interviews. The desire for support to ensure that their unborn children do not acquire the HIV infection was another dominant concern for the women.

For me what came to my mind was how I can protect my child from getting HIV?… The nurse said we should breastfeed our babies, but for me I will not breastfeed… I will try and get cow’s milk for my baby. Questions on how I will keep my self alive to bring up my children keep coming in my mind. Will my children attain education… (Married, 36 years, HIV positive).

From the quote above, a positive HIV test result triggered interest for women to protect their babies from HIV infection but also elicited worries in some women on how to maintain healthy lives and guarantee growth and survival of their children. These findings again show the central place children occupy in the lives of women and how a positive HIV diagnosis brings to the fore the social and economic struggles of women. The worries were even more pronounced among young women whose dreams of seeing their children grow were shattered and the fact that they least expected the positive diagnosis.

For some HIV positive women having children generated the courage to live and ensure that their children grow.

I know I will not die tomorrow, so I have to get treatment and live to see my children grow (Married, 32 years, HIV positive).

For this woman, the knowledge about ARVs gave her hope and determination to live and care for her children. This is also an example of how women understood their experiences of HIV testing within a wider community context where HIV treatment is increasingly becoming available.

Interviews with women who tested HIV negative revealed that most of them got relief from stress and fear; others interpreted the results as a sign of their husbands being faithful and re-enforced the desire to strengthen trust and faithfulness in their marriage as one of the women explained:

For me it was good news. It means me and my husband; we are faithful to each other… I will remain faithful to my husband (Married, 32 years, HIV negative).

### The insufficiency of post-test counselling

Differences between HIV positive and HIV negative women were noted with regard to their views about post-test counselling. Most of the HIV positive women said they were accorded sufficient time during the post-test counselling to receive the results, understand them and think through the next courses of action for themselves and their unborn babies and generally reported that the counselling was satisfactory.

The counselling was adequate. I was given enough time and the counsellor told me to ask any question I wanted to ask (Married, 37 years, HIV positive).

In contrast, some women who tested HIV negative believed they were given limited time during the post-test counselling. As a result some of them went away with unanswered questions and some were not sure whether they were indeed HIV negative.

The time was not enough… the nurse told me I was free from HIV. I have two “hearts”, one “heart” is like it is true I am negative, another is like it is not true… I will test again (Married, 32 years, HIV negative).

Doubts about the HIV negative test results by some women, is a reflection of the limited health worker interaction with such women and constitutes a missed opportunity for HIV prevention. For some HIV negative women, their doubt about the negative HIV results was linked to the supposition that they had syphilis (“Kabotongo”) because they were already experiencing a skin rash, which in their communities was an indication of syphilis, as one woman explained:

For me I have syphilis because I have skin rash and you see how my skin looks like (its whitish)… but the nurse has told me that I do not have HIV and syphilis. If I don’t have it (syphilis) why is my skin like this? … there were many women waiting so I did not ask the counsellor (Married, 23 years, HIV negative).

Interviews with health workers confirmed that some women who test HIV negative indeed doubt their test results:

One time a mother asked me in the room, is it true I am HIV negative? When I asked her why? She said, she had syphilis because of how her skin looked and so she could be having HIV. This woman had attended the group counselling, had tested and received her results but she was not sure. We need enough health workers and to encourage mothers to ask whenever they have a question (Health worker).

What is emerging from the above finding is that women’s doubt about their HIV negative results is influenced by the prevailing community perceptions and beliefs about illnesses like syphilis, but also, the setting characterised by a shortage of health workers negatively affects the quality of HIV counselling, especially for those women who test HIV negative. Some HIV positive women were equally in doubt about their positive test results, especially those who had previously tested HIV negative.

### The conduct of health workers - supportive but constrained

Overall, the health workers were generally described by the women as caring and supportive though overwhelmed by the large number of women they had to attend, which led to limited time to address women’s concerns.

The nurses were good to us. They taught us first in a group and talked to us well. They really care although we are many women who come here. If government can increase the health workers I think they can give us (women) more time (Married, 24 years, HIV negative).

To some HIV positive women the acts of good care by nurses included prayer and providing re-assurance.

The nurse prayed for me and comforted me after telling me my results. Now when I think about that time, the nurse was very good to me. She did a good job… (Married, 28 years, HIV positive).

The above narratives of women indicate that health workers’ ability to provide quality care was limited by lack of staff for a high client load.

## Discussion

The experiences of the women in this study showed that women are apprehensive about HIV testing because of the possibility of a positive HIV result, but the atmosphere created by the health workers has made the women comfortable about testing within the antenatal clinic. At this point, eight years after the introduction of PMTCT at this hospital and four years after introducing routine HIV testing, when the programme has reached maturity, women in general are informed about the procedures before attending. Most women in this study had learned about routine HIV counselling and testing during their past visit to health facilities, through the media and their families and friends, a clear sign of how informal and formal social structures re-enforce each other in creating informed health service users. In part, these findings reveal that women’s acceptance of routine testing is the result of good access to information on the benefits of HIV testing both from informal and formal sources. On the other hand, the general perception by women of routine counselling and testing as ‘compulsory’ for all pregnant women also reinforces women’s acceptance of the test. In general, health workers favoured HIV testing during health education and counselling sessions. In fact, the possibility of not testing (opt-out) was downplayed by the ANC staff to such a point that most women think it is compulsory to test. The apparent acceptance of HIV testing by women could also mirror the inherent power relationships between women and health workers where women may be unable to go against what their health care providers tell them to do. The ongoing discourse, especially in the media, aimed at popularising the PMTCT programme further reinforced women’s perception of HIV testing within the ANC as being compulsory. The perception that HIV testing as part of ANC was compulsory, has also been documented in other African settings [20-22]. The perception by some women that testing is compulsory renders some merit to earlier concerns that RCT might undermine informed consent and patients might feel obliged to test [36]. In the study setting this is however debatable given that most women knew about the test before coming to the health facility and were provided with additional health education within the ANC. Although, there has been speculation that the perceived compulsory HIV testing may negatively affect women’s attendance of ANC, this was not the case in our study setting where the number of new ANC attendees continued to increase even after a shift from VCT to RCT [14].

Women in this study said that taking an HIV test was a difficult step to make. The narratives of pregnant women in this study revealed that they feared a positive test result which was associated with the fear of death, living with HIV and being blamed for bringing HIV infection to the family. However, the routine provision of HIV testing helped such women to overcome the fear and take the test. On the other hand, most women strongly believed that HIV testing was beneficial, especially by enabling those who test HIV positive to protect their babies from HIV infection through enrolling in the PMTCT programme. In this regard, the high acceptance of HIV test could be a reflection of women’s conformity to the moral imperative of doing ‘good’ for their babies. The women also appreciated that HIV testing was beneficial since those found to be positive could access HIV treatment for themselves. The need to protect their children and the concern for their own health have been documented in other African settings as key reasons for women’s acceptance of HIV testing during pregnancy [11,13].

Our findings differ from those of a study done in 2008 in the nearby Iganga district, in Uganda, which found that women did not fully understand the benefits of HIV testing [21]. This difference could in part be explained by differences between the two districts in the initiation of PMTCT programmes (Iganga 2004 and Mbale in 2002) and being different study populations.

Whereas HIV testing was in general perceived as beneficial by women, for some, it placed on them an extra burden. For instance, some women were worried about disclosure of HIV status to their partners and the possibility of being blamed by their sexual partners for bringing HIV infection into the family as has been documented elsewhere [37]. The struggles of living with HIV including uncertainty about the future of their marriage and care for the women and their children were common concerns among HIV positive women in our study. These findings are not surprising given the day to day struggles of women to secure their own and their children’s survival amidst poverty, stigma and marginalisation in the African setting [38]. In the study area, most women, depend on their male partners for their care and that of their children [39].

The benefits of HIV testing for the women who test HIV negative were rarely mentioned by women during interviews. In part, this is a reflection of how HIV counselling has not prioritised the majority of the people who test HIV negative for effective HIV prevention. In line with critical theory, the limited number of health workers in the study setting, as a structural constraint [24], compounded the inadequacy of the post test counselling and support received by HIV negative women. Human resource constraint as a barrier to effective implementation of provider-initiated HIV counselling and testing has previously been documented in Uganda [40] and needs to be re-emphasised.

The women in our study expressed a strong desire to protect their babies from HIV infection as a major motivator for undergoing HIV testing. This strong desire to protect children can be explained by the values attached to children in Uganda even in the time of HIV [41], but also the moral imperative where children are seen as innocent and deserve protection. This strong desire by women to protect their babies has not been taken advantage of by the health system to optimize the PMTCT services in Uganda. Currently, only 51.6% of the estimated HIV positive pregnant women in Uganda receive antiretroviral drugs to reduce the risk of mother-to-child transmission of HIV. This coverage is still insufficient to reach the desired goal of eliminating paediatric HIV infections [42]. However, the strong desire of most women to test for HIV so as to protect their children from HIV infection further complicates the implementation of opt-out option in practice as those who may choose not to test may be perceived or made to feel as ‘not good mothers’ or ‘acting against their own health’. This finding further reveals a real life challenge of making opt-out an option for women.

Some women in our study understood routine HIV testing during pregnancy as a new ‘law’ by the government aimed at protecting babies from HIV infection. For such women, HIV testing can be interpreted to mean embodying the good of the Nation as discussed in another context by Booth Karen [43]. The implication here is that for some women, taking an HIV test may be a reflection of compliance with the state.

Whereas the health education session that doubled as a pre-test counselling session was described by mothers as informative and educative, there was a general need to strengthen post-test HIV counselling for both HIV positive and HIV negative women. A common concern among both HIV positive and HIV negative women was doubt about the test results. Doubt about the positive results among HIV positive women was linked to their limited perception of risk for HIV infection. It could also reflect that they had been faithful to their sexual partners, so they could not imagine how they could have become infected. Such women in doubt may be reluctant to utilize PMTCT drugs or to seek care for their own health.

To our surprise in this study, some women who tested HIV negative did not believe in the results. Doubt about the results among HIV negative women was enhanced when they were told they did not have syphilis, contrary to their locally accepted community ‘diagnosis’ of syphilis based on a skin rash and a pale skin. This is an example of a clash between the socio-cultural definition and understanding of illness and the biomedical understanding of disease. Such women in doubt about their HIV negative sero-status may be reluctant to adopt risk-minimising behaviours, which in turn could have negative effects on fostering primary prevention of HIV as part of the PMTCT programme. This finding depict that the doctor and patient explanatory models often co-exist in a clinic encounter [44] and calls for more collaborative efforts between service users and providers. In general, such apparent contradictions need to be identified and dealt with in health education and counselling. In this case, it is critical that health workers provide sufficient information to pregnant women about syphilis and other conditions that may lead to skin rash and pale skin and how they can be managed. In addition, mothers should be encouraged to re-test if they doubt the results. Another explanation for women’s doubt of their negative HIV status could be linked to the public health messages which tend to over estimate the likelihood of HIV transmission and thus many people often think testing for HIV will lead to a positive HIV result, as has been documented in Malawi [45,46]. Suspicions about partners or reflections about one’s own health and sexual history could be other explanations for doubt of test results.

Health workers were generally described by women as caring and supportive though constrained by being few in number. Indeed, women’s experiences of limited post-test counselling owing to few health workers is a reflection of the national and global human resource crisis affecting many sub-Saharan countries. The limited post-test counselling presents missed opportunities for health workers to foster messages for primary HIV prevention. In Mbale District, the situation is worsened by the intermittent provision of HIV testing services and lack of maternity services at lower level health facilities. Some strategies that may help in this are 1) ensuring that HIV counselling and testing services are regular at lower level health facilities in the district, 2) increasing the number of health workers to allow more time for health worker-client dialogue on the interpretation of biomedical results relating to HIV and other sexually transmitted infections, 3) using lay counsellors who may include persons living with HIV as expert clients, or members of support groups who may also help, especially in providing insights on dealing with the challenges and fears related to a positive HIV diagnosis [47]. The need to improve the quality of HIV counselling as part of PMTCT programmes has been documented by other scholars in Uganda [27] and in Tanzania [48].

Most HIV positive women in this study interpreted their results to mean betrayal and infidelity by their sexual partners. In addition, most of the women who tested HIV positive strongly believed that their partners were already HIV positive and thus not likely to discuss risk reduction strategies such as condom use with their partners. Contrary to this belief, a study of HIV-infected people receiving antiretroviral therapy in Uganda found that 43% of the spouses of HIV-infected married people were HIV negative [49].

Women who tested HIV positive were happy with the immediate counselling, but still expressed long term worries about survival with HIV and the care of their children. Women’s fears and concerns about meeting their needs and those of their children are not surprising given dwindling public services and social support systems in Uganda. In this regard, partnerships with other organisations are needed to address the support and survival of such women.

In contrast, most women who tested HIV negative interpreted the test results as a sign of fidelity of their sexual partners. These findings depict a limited understanding and appreciation among women of the likelihood of discordant HIV test results for themselves and their partners. Moreover, such women may not be motivated to encourage their partners to go for HIV testing. In 2008, it was estimated that 43% of the new HIV infections among adults aged 15–49 years in Uganda were among people in discordant monogamous relationships [3,50]. Thus, the risk for HIV infection even among women who test HIV negative should be emphasized in both pre-test health education and post-test counselling.

The HIV negative women in our study revealed that counsellors advised them to remain faithful to their sexual partners to avoid HIV infection. This is of course good advice but it does not minimize the relevance of the statement that ‘women get HIV in their own bedrooms’, meaning that if only women and not their sexual partners remain faithful, the chance of contracting HIV is still high. More efforts are needed to reach men with HIV testing and HIV prevention messages within and beyond the antenatal clinic.

### Strengths and limitations

Use of qualitative interviews and the inclusion of pregnant women and health care providers in the study facilitated an in-depth understanding of women’s experiences with routine HIV testing. However, our findings should be interpreted in view of the following limitations. 1) The study was conducted at a public hospital in the general antenatal care setting characterized by congestion and delays at the clinic; it is possible that women with higher incomes obtain antenatal care from private clinics and may thus be under-represented in this study. 2) Experiences of women who decline the HIV test could not be gathered since at the time of the study all women who attended the antenatal clinic at the hospital tested. 3) The study was conducted at a regional referral hospital (a tertiary health facility) with experienced health workers and relatively better facilities. Thus the gaps documented in this paper could be more pronounced at remote lower level health facilities with fewer staff and recurrent shortage of HIV test kits 4) Besides, health workers at the study site have been exposed to research for a long time, which may also limit applicability of study findings to other settings with no or minimal exposure to research 5) We conducted all interviews at the health facility since it was not easy to identify pregnant women tested for HIV as part of ANC at community level, especially HIV positive women. This might have biased the respondents to report more of their positive experiences. Indeed, some pregnant women might have been reluctatnt to blame health workers who will eventually help them at the time of giving birth. Besides, women were introduced by health workers to the research team, which might have further enhanced this bias. To minimize information bias, the fact that the researchers were independent of the hospital staff was made explicit to the study participants. In addition, qualitative interviews involving use of probes and triangulation of data from different sources helped to improve the trustworthiness of our findings.

## Conclusions

In this setting, routine counselling and testing services are known and acceptable to mothers. There is need to strengthen post-test and follow up counselling for both HIV positive and negative women to maximize opportunities for primary and post exposure HIV prevention. Partnerships and linkages with people living with HIV, especially those in existing support groups such as those at The AIDS Support Organization (TASO), may provide further post-test counselling and support for the pregnant women. This could take the form of helping women at Mbale Hospital to start post-test support groups or involving expert clients from TASO in antenatal-based health education talks at the hospital. For effective HIV prevention, women who test negative should be supported to remain negative. More efforts to reach men with HIV testing and prevention messages, including discussions on fidelity, are needed within and beyond the antenatal clinic.

## Competing interests

The authors declare that they have no competing interests.

## Authors’ contributions

JR conceived the study. All authors (JR, SN, JKT, TT, & HKH) participated in the design of the study. JR participated in data collection. JR and HKH participated in data analysis. JR wrote the initial draft of the manuscript. All authors (JR, JKT, TT, SN & HKH) participated in the interpretation, revision of manuscript and approval of the final manuscript.

## Pre-publication history

The pre-publication history for this paper can be accessed here:

http://www.biomedcentral.com/1472-6963/13/189/prepub
